# Wandering ST-Segment in Acute Coronary Syndrome: The Einthoven’s Twist

**DOI:** 10.7759/cureus.50089

**Published:** 2023-12-06

**Authors:** Bharath Raj Kidambi, Sriram Veeraraghavan, Soorampally Vijay

**Affiliations:** 1 Cardiology, All India Institute of Medical Sciences, New Delhi, Delhi, IND; 2 Cardiology, SRM Medical College Hospital and Research Center, Chennai, IND; 3 Cardiology, Trilife Hospital, Bengaluru, IND

**Keywords:** anterior wall acute myocardial infarction, inferior wall myocardial infarction, occlusion mi, electrocardiogram (ecg/ekg), coronary artery vasospasm, lead misplacement, wandering myocardial infarction

## Abstract

Interpretation of the ST-segment axis in ST-elevation myocardial infarction (STEMI) plays a crucial role in identifying the culprit artery and optimizing revascularization strategies. In certain conditions, the ST-segment axis may abruptly change during management, creating diagnostic confusion, provoking unnecessary workups, and causing treatment delays. Some reported causes of wandering ST-segment include lead misplacement, progressive injury, coronary vasospasm, migration of the thrombus, and aortic dissection. Here we describe two exciting cases of wandering ST-segment axis in acute coronary syndrome and its management.

## Introduction

The ST-segment elevation in electrocardiogram (ECG) is essential in diagnosing and optimizing therapeutic strategies in ST-elevation myocardial infarction (STEMI) by determining the presence, location, and extent of jeopardized myocardium. Occasionally, there occurs a phenomenon of “wandering ST-segment elevation” characterized by ST-segment elevations seen in some leads initially, which disappear and reappear in different leads. It may lead to diagnostic confusion and treatment delays or provoke unwanted procedures at times. Here we describe two such cases of wandering ST-segment elevations caused by incorrect lead placement in one and progressive injury expansion in another.

## Case presentation

Case 1

**Figure 1:**
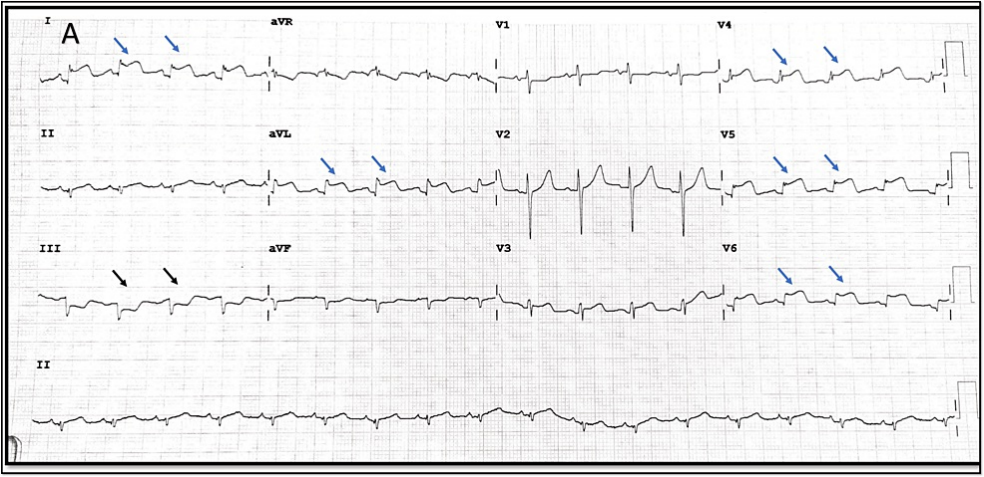
12-lead ECG on admission ST-segment elevation seen in leads I, aVL, V4-V6 (blue arrow) with reciprocal depression in inferior leads (black arrow)

A middle-aged lady presented to the emergency department with complaints of progressive breathing difficulty for 10 hours with associated sweating and giddiness. A general physical examination revealed an anxious patient with a blood pressure of 90/60 mmHg, a heart rate of 108/minute, a respiratory rate of 32/minute, body temperature of 37 degrees Celsius, and an oxygen saturation of 92% at room air. The physical examination revealed an anxious and dyspneic patient with audible crepitations till mid-thorax who was immediately started on oxygen therapy, vasopressor support, and intravenous diuretics. Past medical history was significant for poorly controlled type 2 diabetes mellitus for 8 years. Suspecting acute coronary syndrome, an ECG was done, which revealed ST elevation in anterolateral leads (leads I, AVL, V4-V6) with reciprocal changes noted in lead III (Figure 1).

**Figure 2:**
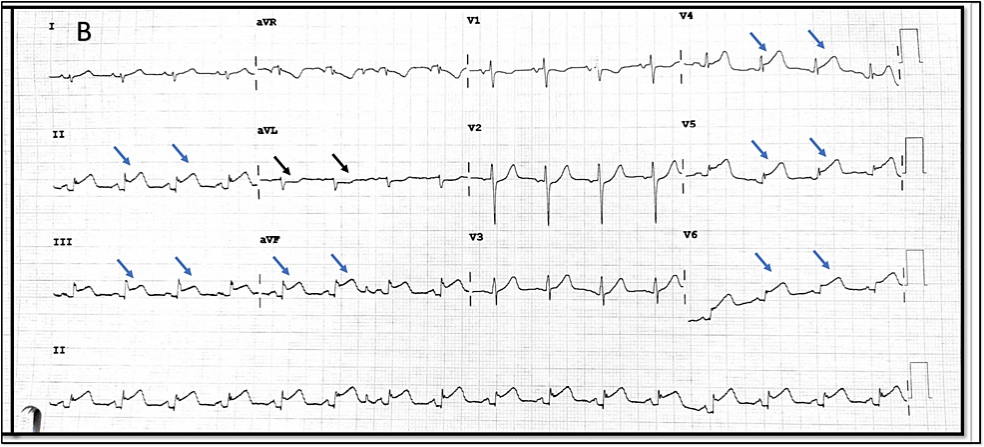
12-lead ECG - taken subsequently 12-lead ECG showing wandering ST-elevation from lead I, aVL in Figure 1 to involve leads II, III, aVF (Blue arrow) now. Reciprocal ST-depression seen in lead aVL (black arrow)

She was quickly given loading doses of ticagrelor 180 mg, aspirin 300 mg, and atorvastatin 80 mg, and the patient was being stabilized with a plan for being taken up for primary PCI. Preliminary investigations revealed a troponin of >10 ng/ml of troponin I (n < 0.04 ng/ml), with capillary blood glucose levels of 22 mmol/L, positive urine ketones, and metabolic acidosis (pH 7.2). An insulin infusion was started for the management of diabetic ketoacidosis. After stabilization, another ECG was repeated in 10 minutes, which revealed no ST elevation previously seen in leads I and aVL but new ST-Segment elevations in leads II, III, and aVF. Left precordial leads showed persistent ST-segment elevation (Figure 2).

**Figure 3:**
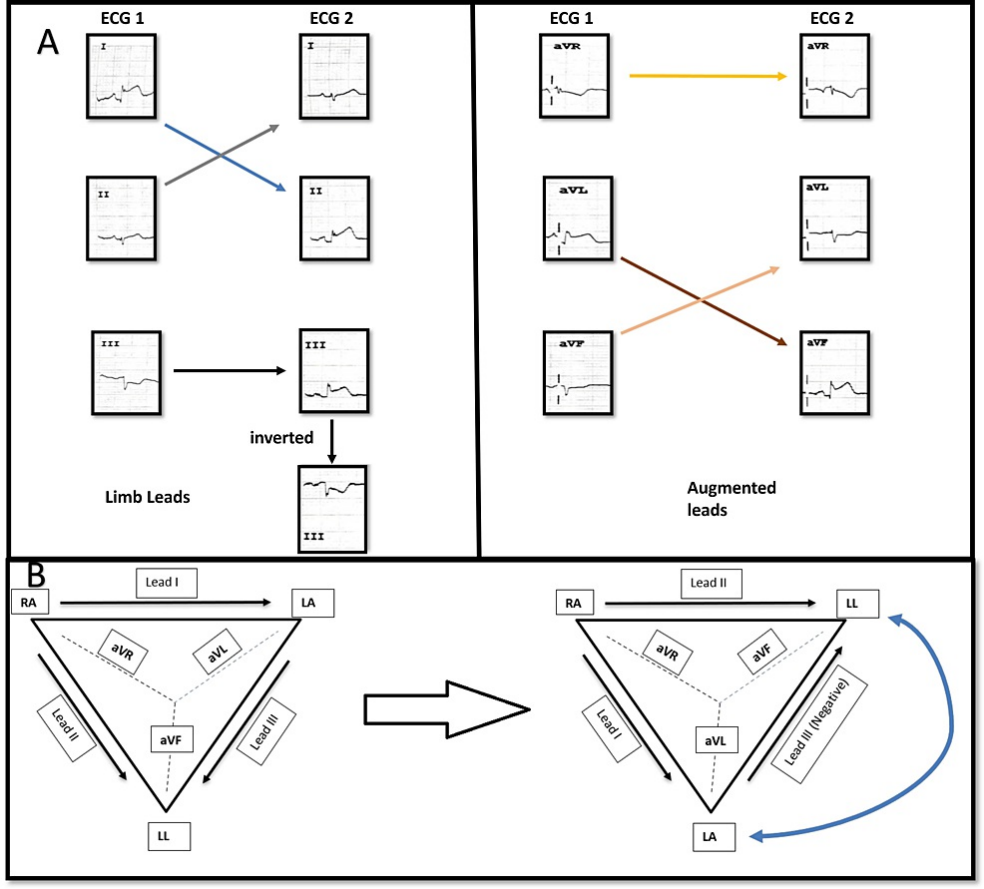
Comparison of ECG on admission to subsequent ECG A) Comparison of limb leads and augmented leads in 1st and 2nd ECG, showing interchange of leads I and II, aVL and AVF, inversion of lead III. The lead aVR remains the same; B) Einthovenʼs triangle showing vectors of leads in normal conditions and in limb lead reversal.

The initial diagnosis of anterior wall MI was based on the clinical picture and ECG findings. Upon close examination of both ECGs, axis changes were noted only in limb leads and augmented leads. There were no discernible changes in precordial leads, which led us to suspect incorrect lead placement. There was a 180-degree vertical rotation around the axis of aVR, with the switching of leads I and II, aVL and aVF, and the inversion of lead III (Figures 3A, 3B).

**Figure 4:**
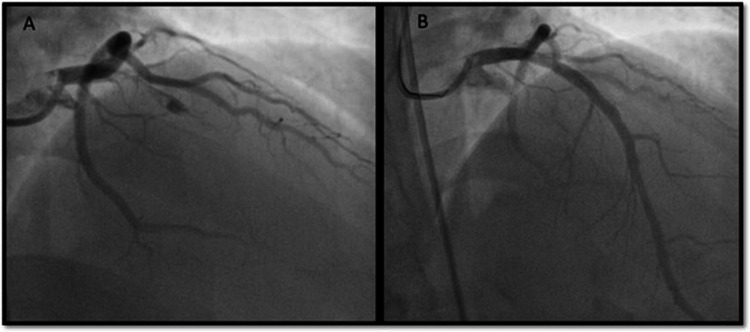
Angioplasty findings Fluoroscopic coronary angiography images showing thrombotic occlusion of the left anterior descending artery (A) and good flow established after primary angioplasty (B)

Echocardiography showed hypokinesia in the basal and mid-anterior and lateral walls, normal wall movement in the inferior territory with moderate LV systolic dysfunction, and an ejection fraction of 38%. Diagnostic angiography showed a thrombotic complete occlusion in the left anterior descending artery, with no lesion in the right coronary artery confirming our suspicion of lead misplacement. Primary angioplasty of the culprit vessel left anterior descending artery (LAD) with a 3.5 x 28 mm drug-eluting stent (DES) with a good end result (Figure 4A, 4B).

Case 2

**Figure 5:**
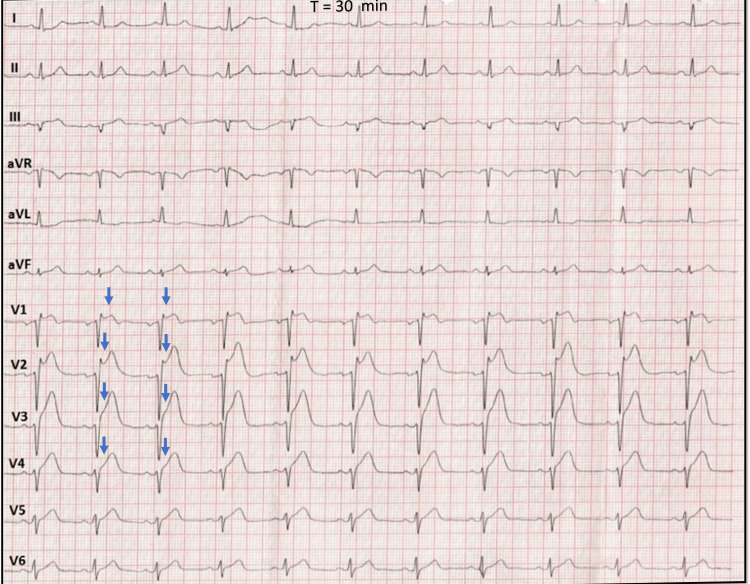
12-lead ECG on referral 12-lead ECG from the referring hospital, showing concave-up ST-segment elevation in leads V1-V4 (blue arrow) at 30 minutes duration since chest pain.

A 65-year-old gentleman with no prior comorbidities had presented to the nearby primary healthcare center in the early morning with typical chest pain of approximately half an hour duration. Initial evaluation revealed an agitated patient having profuse sweating, with a blood pressure of 100/60 mmHg in the right arm, heart rate of 55/minute, respiratory rate of 28 per minute, maintaining a saturation of 98% on room air. The clinical examination was significant for elevated jugular venous pressure. The lungs were clear on auscultation. The electrocardiogram taken showed predominant ST-Segment elevation in leads V1-V4 (Figure 5).

**Figure 6:**
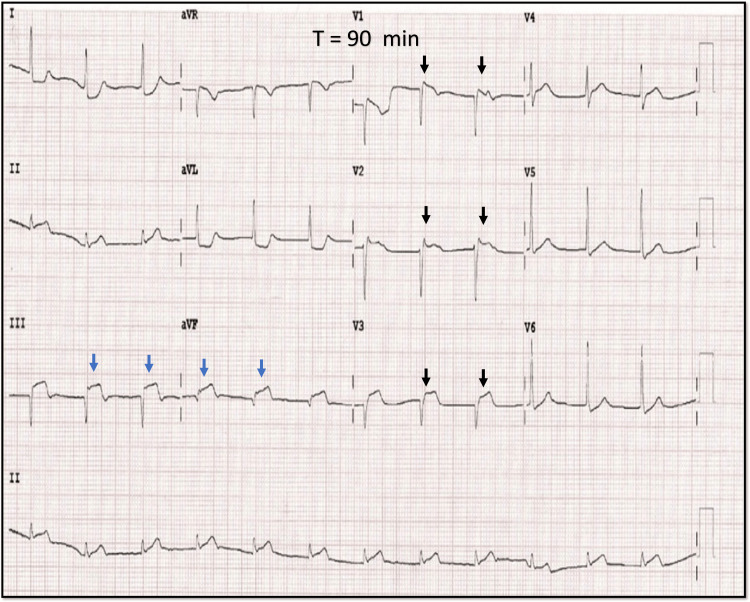
12-lead ECG at our ER 12-lead ECG at our center showing decreased amplitude of ST-segment elevation in precordial leads seen previously (V1-V4) (black arrow). ST-segment elevation is prominent in inferior leads III, and aVF (blue arrow). Reciprocal ST-segment depressions were noted in leads aVL and lead I.

He was given a loading dose of 325 mg of aspirin, 600 mg of clopidogrel, 80 mg of atorvastatin, and one dose of sublingual glyceryl trinitrate for his pain and was referred to our tertiary care center, which is 1 hour away, with a diagnosis of anterior wall myocardial infarction for primary angioplasty. Upon arrival at our ED, the patient looked drowsy, with a blood pressure of 70/50 mmHg in the right arm. Blood pressure was stabilized after a quick bolus of normal saline and norepinephrine drip. Initial labs revealed mild metabolic acidosis with a pH of 7.30, troponin I was elevated at >10 ng/ml, and lactate was elevated at 3 mmol/l. The blood sugars and electrolytes were within normal limits. After initial stabilization, another ECG was taken (Figure 6) prior to shifting the patient to the cardiac catheterization lab.

**Figure 7:**
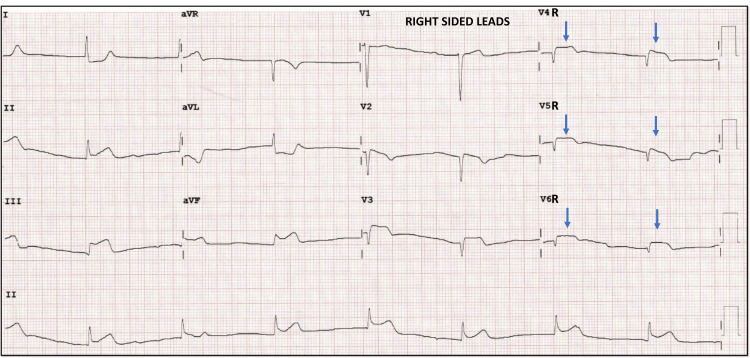
12-lead ECG - right-sided leads 12-lead ECG, right-sided leads showing bradycardia, junctional rhythm, ST elevation in VR4-to V6R (blue arrow) suggestive of proximal RCA occlusion. RCA: right coronary artery

To our surprise, there was a decrease in the amplitude of ST-segment elevation seen previously in V1-V3, with a new ST-segment elevation seen in inferior leads III, aVF. There is bradycardia, with a junctional rhythm, and ST elevation in right-sided leads (Figure 7) suggestive of the inferior wall with right ventricular MI probably due to occlusion in the proximal right coronary artery.

**Figure 8:**
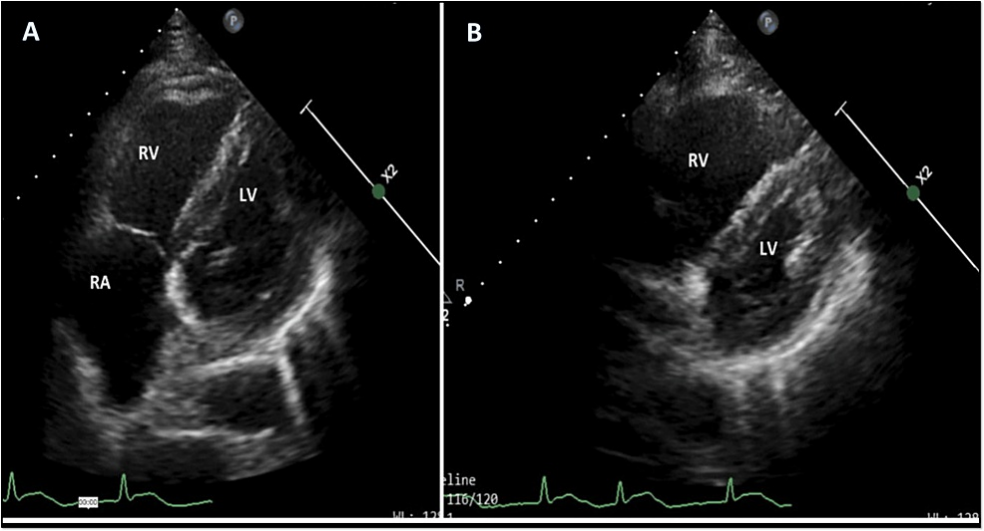
Transthoracic echocardiography Echocardiography showing RA, RV dilatation (A), and D-shaped septum (B) RA: right atrium, RV: right ventricle

Patient credentials and validity of initial ECG were confirmed with the referring hospital. Differential diagnoses at this stage included progression of transmural injury, coronary vasospasm, aortic dissection, incorrect lead placement, and wrap around the left anterior descending artery. Echocardiography showed dilatated right atrium and right ventricle (RV) with a “D”-shaped septum, severe RV dysfunction, and hypokinesia in the inferior wall of the left ventricle (Figures 8A, 8B).

**Figure 9:**
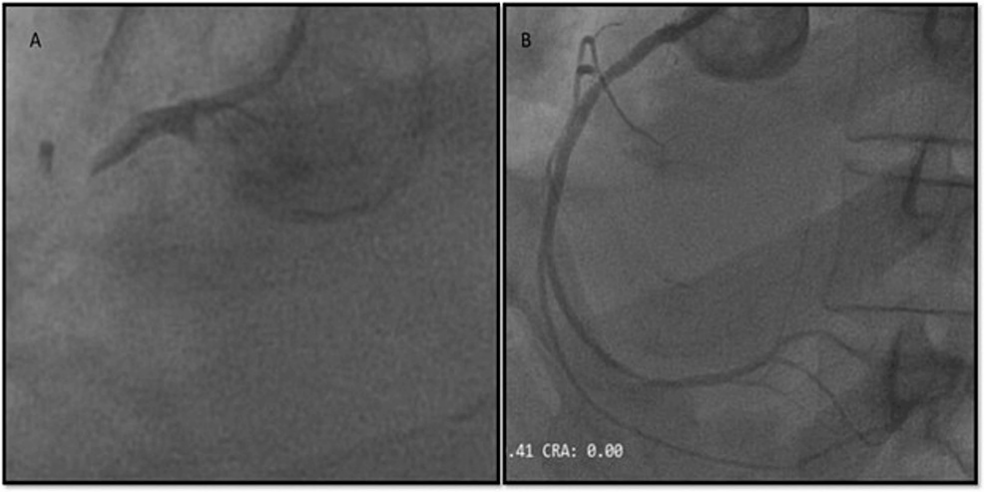
Angioplasty images Fluoroscopy coronary angiography images showing complete thrombotic occlusion of RCA (A) and after subsequent revascularization (B)

The coronary angiogram revealed a total thrombotic occlusion of proximal RCA prior to the conal branch, and a 3.00 x 38 mm DES was deployed to achieve a good end result (Figures 9A, 9B).

The other arteries were normal excluding the other differential diagnosis. The patient subsequently recovered from the cardiogenic shock and is now doing well on follow-up.

## Discussion

**Figure 10:**
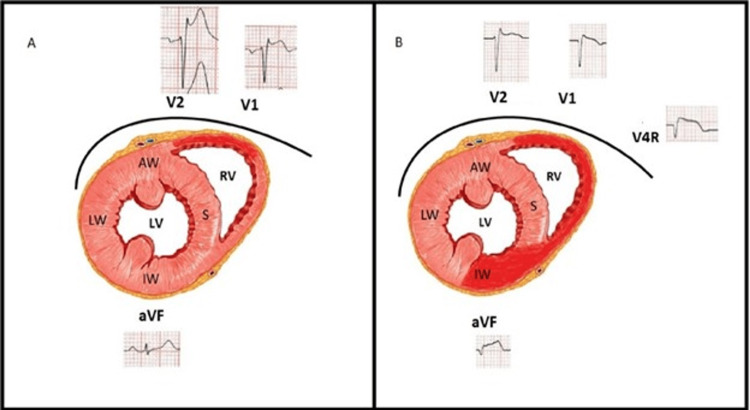
Diagrammatic representation Diagrammatic representation depicting the correlation of ECG changes in anterior and inferior leads at 30 minutes and 90 minutes.

The conventional 12-lead ECG plays a central role in the therapeutic decision pathway in patients with chest pain [1]. According to the ACA/AHA criteria, diagnosis of STEMI is done when there is a new ST-segment elevation at the J point in at least two contiguous leads of ≥ 2 mm (0.2 mV) in men or 1.5 mm (0.15 mV) in women in leads V2-V3 and/or of ≥ 1 mm (0.1 mV) in other contiguous chest leads or the limb leads. There are also special algorithms to predict the culprit artery based on the leads showing the ST-Segment elevation [2,3]. In rare circumstances, ST-segment elevation may appear to wander from one lead to another during the management of ACS and cause diagnostic and therapeutic dilemmas [4-6]. Our first patient had wandering ST-segment elevation due to incorrect lead placement, a commonly encountered but underreported issue. Incorrect lead placement between the right arm - left arm electrodes occurs frequently and is identified by the extreme QRS axis, with the R wave in lead aVR. However, in isolated left arm left leg lead reversal, the aVR lead remains unchanged with a significant alteration in the vector of other leads [7]. If unrecognized, it may lead to further tests being ordered, causing a delay in revascularization. Our case reiterates the importance of suspecting incorrect lead placement as an important differential in wandering ST-segment elevation. Our second patient with RCA occlusion presented early (T = 30 min) showing ST elevation initially only in left precordial leads (V1-V3), reflecting probably the injury vector directed toward an anteriorly placed and thin-walled RV [8]. The second ECG taken after the transfer (T = 90 min) showed that the ST-segment elevation had wandered to involve inferior leads reflecting a transmural injury to the entire RV and progression to involve the inferior wall of LV (Figures 10A, 10B).

The initial pattern of ST-elevation mimics anterior wall involvement, and in such scenarios, certain ECG clues may predict RCA’s involvement over LAD. These are decremental ST elevations from V1-V4, ST elevations in right-sided leads (V3R, V4R), and the absence of Q wave in V1-V3 following ST resolution. The magnitude of ST elevation during RV involvement is usually higher in V1 compared to other leads [9]. However, it depends on the patient’s body geometry, body habitus, and degree of counterclockwise rotation, which may be possible explanations for V2 showing the maximum elevation in our patient. This case highlights two important points. The first is to consider RCA occlusion in the differential diagnosis for any ST elevation involving leads V1-V3, and the second is progressive injury as a probable cause of wandering ST-segment elevation.

## Conclusions

A few salient aspects are brought to light in both our cases. During acute coronary syndrome, it is critical to recognize a few key differentials for wandering ST-segment elevation, such as lead misplacement, coronary vasospasm, infarct expansion, and thrombus migration. ECG electrode misplacements are frequent and underreported; it is important to rule out lead misplacement before proceeding to further evaluation. The second key learning point is to keep right coronary artery infarct in the differential diagnosis of ST-elevation of leads V1-V3.
